# The effectiveness of educational program based on health belief model on promotion of puberty health concepts among teen girls: a cross-sectional study in north of Iran

**DOI:** 10.1186/s12905-023-02391-3

**Published:** 2023-05-08

**Authors:** Sedighe Bab Eghbal, Zahra Aghaei Kenari, Asieh Ashouri, Nooshin Rouhani-Tonekaboni, Parisa Kasmaei, Fardin Mehrabian, Mahmood Karimy, Fatemeh Rezaei, Esmaeil Fattahi

**Affiliations:** 1 Reproductive Health Research Center, Department of Obstetrics and Gynecology, Al-Zahra Hospital, School of Medicine, Guilan University of Medical Sciences, Rasht, Iran; 2 School Health Expert of University Health Vice Chancellor, Guilan University of Medical Sciences, Rasht, Iran; 3 Cardiovascular Diseases Research Center, Department of Cardiology, School of Medicine, Department of Health Education and Promotion, Heshmat Hospital, Guilan University of Medical Sciences, Rasht, Iran; 4 Research Center of Health and Environment, Department of Health Education and Promotion, School of Health, Guilan University of Medical Sciences, Rasht, Iran; 5 Research Center of Health and Environment, Department of Health Education and Promotion, School of Health, Guilan University of Medical Sciences, Rasht, Iran; 6 Department of Public Health, Social Determinants of Health Research Center, Saveh University of Medical Sciences, Saveh, Iran; 7 Adolescent Health Expert of University Health Vice Chancellor, Guilan University of Medical Sciences, Rasht, Iran; 8 Department of Health Education and Promotion, School of Health, Guilan University of Medical Sciences, Rasht, Iran

**Keywords:** Education, Puberty, Health belief model, Girl

## Abstract

**Background:**

Puberty is a sensitive critical stage of human life. As numerous healthy habits and behaviors are created during adolescence, correct health education during puberty is essential to maintain and improve an individual's physical, emotional, and mental health. The present study aimed to determine the impact of educational intervention based on the predictors of the Health Belief Model (HBM) on female nine-grade students’ health behaviors in Rasht, Iran.

**Methods:**

The present randomized controlled trial study examined 110 female nine-grade students. Multi-stage sampling was performed, and the students were randomly divided into two groups of 55 as intervention and control. The data collection tool included a valid and reliable questionnaire with four sections, namely demographic variables, knowledge, HBM constructs, and health behaviors during puberty. The educational program comprised four 45–60-min sessions per group (4 groups of 13) based on HBM. The data were collected two times, before and 1 month after the educational intervention, and were analyzed using the independent t-test, paired t-test, chi-square test, and SPSS 23.

**Results:**

The mean age of menarche was 12.26 ± 1.133 in the intervention group and 12.12 ± 1.263 in the control group. The family was a source of information for students and the main cue to action before the intervention. Before the educational intervention, there was no significant difference between the experimental and control groups in terms of knowledge, HBM constructs, and puberty health behaviors; however, the variables increased significantly in the intervention group after educational intervention (*P<*0.001).

**Conclusions:**

Given the effectiveness of the HBM in improving the health behavior of adolescent girls, it is recommended that health policymakers should plan and implement educational interventions in this field.

## Background

Adolescence is an important sensitive stage of human life that all individuals' experience. It is a bridge between childhood and adulthood when puberty occurs as a turning point of adolescent changes. Puberty is considered an underlying period for different stages of life. This period of life is crucial in terms of health as most individual health habits are created in this stage, having a considerable impact on health behaviors in adulthood [1, 2]. Sufficient knowledge regarding the natural process and problems of puberty leads to adolescents' successful and healthier passage from this stage [3, 4].

Lack of education, wrong education, embarrassment, and avoiding discussion about genital health prevent adolescent girls from achieving mental and social health, so that they will have no positive feelings about themselves and their abilities, causing numerous problems [5]. Girls and women were inhibited to eat certain foods and bath during menstruation in countries like Nepal. Women, who worked in clothing factories in Bangladesh, used scraps of cloth as menstrual pads, and many rural African schools had no toilets and water; hence, girls had to stay home during menstruation and missed school exams [6]. For cultural reasons in Iran, most adolescents, especially girls, have no appropriate and sufficient information regarding the physical and mental changes of puberty. Hence, obtaining information from unreliable and uninformed sources causes physical and mental problems for adolescents as only 46.6% of them mentioned mental, physical, and social growth as characteristics of puberty, almost half of them considered only menstruation as a physical change during puberty, and 12.2% considered menstruation as a disease. Furthermore, only 53% bathed during menstruation and 37.1% washed after defecation during this period [7, 8].

Choosing a health education model is the first step in the educational planning process as it starts the program on the right path and maintains it in the right direction [9]. The Health Belief Model (HBM) is an important and accurate model for health-related behaviors and a key model for the development and design of prevention programs [10]. Given the complex and real relationships of attitudes, beliefs, and behaviors, the health education and promotion model, HBM, is used for health promotion and preventive behaviors [11]. Despite the importance of puberty, few studies have investigated this field. Since many health habits and behaviors are formed during adolescence, proper health education during puberty is necessary [12]. Choosing effective, cheap, accessible, and comprehensive educational solutions to improve the health of adolescents is a major concern of health policymakers around the world. Therefore, theory-based research in diverse populations and cultures opens new avenues for social psychologists interested in youth development, health, and education [13]. Based on the results of one study on girls' puberty health in Tehran, perceived benefits and barriers were the most important predictive constructs [14] The results of two intervention studies based on HBM in adolescent female students indicated the effectiveness of HBM-based education in adopting puberty behaviors, thereby promoting maturity and improving the physical performance and perception of the individuals [15, 16]. It should be noted that a similar study has not been conducted in Guilan province. In this research, different educational methods and equipment were used for each construct. Efforts were made to ensure that the educational intervention sessions were based on the culture of the society and the interests of the students. According to the plan, the organizers of the meetings were the students themselves. The present study aimed to investigate the effect of HBM-based educational intervention on puberty health in female nine-grade students at public schools of Rasht, Iran.

## Materials and methods

### Design

The female nine-grade grade students of public schools in Rasht participated in a quasi-experimental study, which was approved by the Ethics Committee of Guilan University of Medical Sciences with an ethical code IR.GUMS.REC.1397.240 participated from September to December 2020. The research population consisted of 110 students (two groups of 55 as intervention and control). The sample size was obtained 44 according to one article by Kazemi et al. [16], and α = 0.05 and ß = 0.10, as well as the following equation. Considering a probability of a 20% drop, the number of students for this study was estimated to be 55 per group.

$$\begin{gathered} n = \frac{{\left( {Z_{1 - \alpha /2} + Z_{1 - \beta } } \right)^{2} \left( {\sigma_{1}^{2} + \sigma_{2}^{2} } \right)}}{{d^{2} }} \hfill \\ \begin{array}{*{20}c} {} & = \\ \end{array} \frac{{\left( {1.96 + 1.28} \right)^{2} \left( {11.32^{2} + 9.24^{2} } \right)}}{{(53.94 - 46.82)^{2} }} = 44 \hfill \\ n^{\prime} = \frac{n}{1 - f} = \frac{44}{{1 - .1}} = 49 \hfill \\ \end{gathered}$$

### Samples and setting

**Fig. 1:**
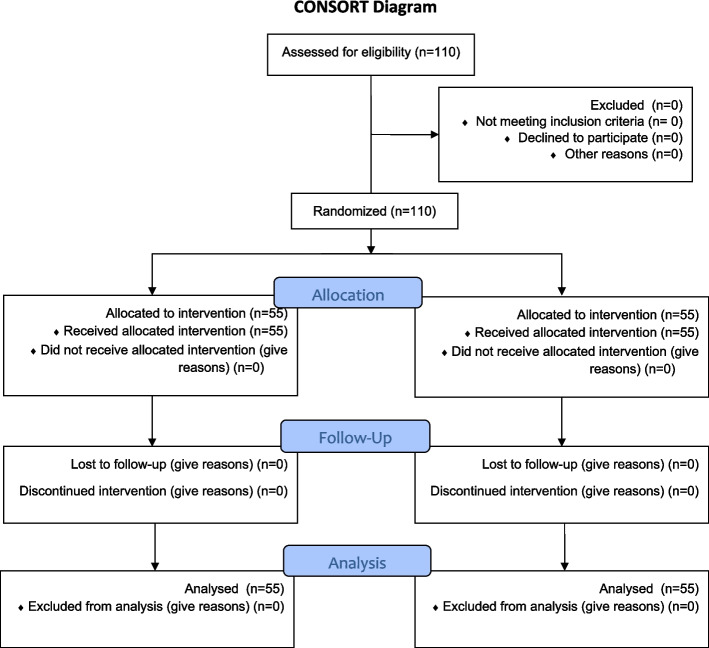
Consort diagram

The multi-stage sampling method was performed. First, a list of public schools in different regions of Rasht was prepared, and then, 4 schools were randomly selected and assigned to control and intervention groups (two schools for the intervention group, and two schools for the control group). The schools were randomly selected from one group at a far distance from each other to prevent the transfer of information between the control and intervention groups. One class from each school was randomly selected, and all students in the class were included in the study. The flowchart Consort drawn (Fig. 1). Inclusion criteria: Studying in the nine grades; having an experience of menarche (first menstruation), and having consent to participate in the study.

Exclusion criteria: Incomplete questionnaire; and non-participation in at least one educational session.

### Measures

The data collection tool was one questionnaire by Kazemi et al. and Shirzadi et al. [15, 16]. To obtain the questionnaire validity, the tool was examined by seven faculty members and research experts (three health specialists, two obstetricians, and two statisticians and epidemiologists). The content validity ratio (CVR) was measured to examine the necessity of the items, and the content validity index (CVI) was calculated to examine three criteria, namely the simplicity, specificity, and clarity of the items. The CVR score was obtained 1, and the CVI score was 1–0.83 for each question or for the average questions, indicating the validity of the questionnaire. Cronbach's alpha coefficient was used to determine the reliability of the questionnaire. A total of 25 students completed the questionnaires (except for the groups participating in the study). Cronbach's alpha coefficient was 0.75, 0.76, 0.81, 0.86, 0.76, 0.78 and 0.88 for knowledge questions, perceived sensitivity, severity, benefits, barriers, self-efficacy, and performance, respectively. Accordingly, the reliability of the tool was confirmed.

The questionnaire consisted of 1- demographic characteristics (15 questions), 2- questions about knowledge (25 questions), 3- questions about the health belief model constructs (42 questions), and questions about performance or behavior (20 questions). Scoring the knowledge questions was in a way that a correct answer was scored 2, a wrong answer was scored 0, and the "I don't know option" answer was scored 1. A higher score indicated greater knowledge. Questions about HBM constructs included perceived sensitivity (*n* = 5), perceived severity (*n* = 8), perceived benefits (*n* = 6), and self-efficacy (*n *= 8) based on a 5-point Likert scale of 1–5 (strongly agree, agree, neutral, disagree, and strongly disagree). A higher score indicated a high level of HBM construct and a probability of adopting the preventive behavior. Perceived barriers (7 questions) were scored on a 5-point Likert scale (strongly agree, agree, neutral, disagree, and strongly disagree) from 1 to 5. Therefore, a lower score indicated lower levels of perceived barriers and the probability of adopting preventive behavior. For performance questions (n = 20), correct behavior was scored 1, wrong behavior was scored 0, and higher scores indicated better performance. The questionnaires were completed by the students, and they were assured that they could leave the study at any time, and their participation in the study would be voluntary. The data were analyzed by the SPSS (ver. 22) software.

The educational intervention was performed directly based on the HBM constructs and educational methods proportional to them by the project executive using various methods (Table 1). The students (intervention group) were first classified as four groups of 12 for the educational intervention purpose, and then 4 educational sessions (four 45–60-min educational sessions) were considered for each group. Data collection was performed in two stages, before the intervention and one month after the intervention, for both groups [12].

**Table 1:** Details of the educational content based on HBM

**Educational sessions**	**HBM constructs**	**Teaching method**	**Objectives**	**Session content**	**Materials and teaching aids**
Session 1	Knowledge	Lecture, Slides, Asking/answering questions, Group discussion	- Increasing the students' knowledge about the present research - Increasing the knowledge about puberty and the importance of complying with health tips during adolescence	- Physical changes during puberty - Menstruation - Complying with personal health during puberty - Health recommendations for this period - Bathing - Exercise - Nutrition - Ways to prevent iron deficiency - Ways to prevent stomach bloating - Acne - Recommendations to reduce pain - Time to visit the physician	Slides, Pamphlet, Video projector, Whiteboard
Session 2	Perceived sensitivity	Lecture, Slides, Asking/ answering questions	Sensitizing individuals to the importance of puberty health - The seriousness of complications and problems of not observing puberty health	Preventive behavior about puberty health, physical, psychological-emotional consequences, Negative consequences of not observing puberty health on girls' health and childbearing	1- Pamphlet, 2- Video projector, 3- Slides, 4- Whiteboard
Session 3	Perceived barriers	Group discussion, Brainstorming	Improving the students' levels of perceived benefits by observing puberty health - Detecting personal and familial barriers - Finding solutions to decrease the perceived barriers	Risk factors and ways to prevent them - The benefits and importance of performing healthy behaviors during puberty - Ways to increase the ability to successfully perform health behaviors	1- Video projector, 2- Whiteboard, 3- Slides
	Perceived benefits	Lecture, Group discussion, Brainstorming, Using motivations			
Session 4	Perceived self-efficacy	1- Lecture with slides, 2- Asking/ answering questions 3- Practical demonstration, 4- Interviews with a believable role model	Increasing perceived self-efficacy for the successful implementation of health behaviors during puberty	Interviews with successful people - Verbal encouragement and persuasion - Decreasing unpleasant feelings and increasing the feeling of happiness -Observing the behavior of successful people	1- Video projector, 2- Whiteboard 3- Slides, 4- Educational video

### Statistical analysis

The central indices, dispersion, and absolute and relative frequency distribution indices were used to describe the study data. The data normality was examined by skewness indices and the Kolmogorov–Smirnov test. A comparison of variables between the two groups was performed using the independent t-test and Mann–Whitney U test. Furthermore, the paired t-test and analysis of covariance were employed to compare the adjusted averages of each index between the two groups after the intervention. The hypotheses were performed bilaterally in all cases, and probability values less than 0.05 were considered statistically significant.

## Results

A total of 110 research units (55 in the intervention group and 55 in the control group) participated in the educational intervention. Most of the students participating in the research were the first children in the family and they knew about menstruation before age of menarche; hence, they were not afraid of their first menstruation. Furthermore, there was no statistically significant difference between the two groups (*P* < 0.52). Both groups were similar in terms of demographic variables and had no statistically significant difference (*P* > 0.05) (Table 2). There was also no significant difference between the intervention and control groups in terms of age, puberty age (age of first menstruation), and mother's age of most of the students participating in the research (*P* > 0.22) (Table 3).

**Table 2:** Comparison of qualitative variables in two groups of intervention and control students

**Variable**	**Levels**	**Intervention group**		**Control group**		**Chi-square Tests**
		**Frequency**	**(%)**	**Frequency**	**(%)**	
**Birth order**	1	34	58.6	30	52.6	*P*-value 0.832
	2	18	31	18	31.6	
	3 and higher	6	10.3	9	15.8	
**Father’s job**	Employee	17	29.3	16	28.1	*P*-value 0.770
	Self-employed	34	58.6	33	57.9	
	Retired	7	12	8	14.1	
**Mother’s job**	Employee	7	12.1	6	10.5	*P*-value 0.520
	Self-employed	5	8.6	6	10.5	
	Retired	2	3.4	0	0	
	Housewife	42	72.4	45	78.9	
**Mothers’ level of education**	Primary school	5	8.6	3	5.3	*P*-value 0.457
	Guidance school	7	12.1	9	15.8	
	High school and diploma	24	41.4	30	52.6	
	University	19	32.8	15	26.3	
**Economic status**	Weak	1	1.7	3	5.3	*P*-value 0.370
	Moderate	32	55.2	24	42.1	
	Good and excellent	24	41.3	30	52.6	
**Information about menstruation before menarche**	Yes	49	84.5	40	70.2	*P*-value 0.067
	No	9	15.5	17	29.8	
**Fear of menstruation**	Yes	21	36.2	26	45.6	*P*-value 0.305
	No	37	63.8	31	54.4

**Table 3:** Age, age of puberty (first menstruation) and mother's age of students in two groups (intervention and control)

**Variable**	**Intervention group**	**Control group**	**Chi-square Tests**
	**Mean ± SD**	**Mean ± SD**	
**Age of students**	14.62±0.489	14.70±0.597	*P*-value 0.221
**Age at puberty (first menstruation)**	12.26±1.133	12.12±1.263	*P*-value 0.896
**Mother's age**	41.9±4.787	40.75±4.937	*P*-value 0.361

According to Table 4, the mean scores of knowledge, perceived sensitivity, perceived severity, perceived benefits, perceived barriers, and perceived self-efficacy were not significantly different in the intervention and control groups before the intervention (*P* > 0.05). There was a significant increase in the scores of all model constructs and behavior in the intervention group after the intervention (*P* < 0.001). Nevertheless, the scores of knowledge, perceived sensitivity, and behavior were not significant in the control group before and after the intervention (*P* > 0.05). The difference was statistically significant for the other constructs before and after the intervention (*P* < 0.05); however, the difference in the means was slight. Data before the educational intervention indicated that the family as a source of information for students had the highest frequency (72.4%) (Table 5).

**Table 4:** Comparison of HBM constructs in two groups at before and after of intervention

**Variable**	**Levels**	**Intervention group**		**Control group**		***P*-value**
		**Mean**	**SD**	**Mean**	**SD**	
**Knowledge (attainable range:0-50)**	Before intervention	31.43	4.60	28.96	5.01	0.07
	After intervention	37.57	4.19	29.12	4.89	< 0.001
	Score change	6.14	5.07	0.16	0.77	
	*P*-value	< 0.001		0.129		-
**Perceived susceptibility (attainable range:5-25)**	Before intervention	18.22	3.009	17.58	3.26	0.27
	After intervention	22.03	1.955	17.46	3.29	< 0.001
	Score change	1.01	2.55	-0.12	0.50	
	*P*-value	< 0.001		0.070		
**Perceived severity (attainable range:8-40)**	Before intervention	28.07	3.29	27.46	3.59	0.34
	After intervention	34.22	2.72	27.23	3.66	< 0.001
	Score change	1.47	3.33	-0.23	0.63	
	*P*-value	< 0.001		0.008		
**Perceived benefits (attainable range:6-30)**	Before intervention	20.98	2.59	20.35	2.71	0.20
	After intervention	26.12	2.27	20.05	2.65	< 0.001
	Score change	1.84	2.92	-0.29	0.68	
	*P*-value	< 0.001		0.002		
**Perceived barriers (attainable range:7-35)**	Before intervention	16.88	3.01	17.30	4.09	0.53
	After intervention	13.28	2.29	16.86	4.04	< 0.001
	Score change	-1.42	3.63	-0.44	0.82	
	*P*-value	< 0.001		< 0.001		
**Perceived self‑efficacy (attainable range:8-40)**	Before intervention	27.96	5.12	27.26	5.43	0.48
	After intervention	36.07	2.40	26.95	5.56	< 0.001
	Score change	2.11	3.89	-0.32	0.76	
	*P*-value	< 0.001		0.003		
**Puberty health behaviors (attainable range:0-20)**	Before intervention	14.90	2.89	14.66	2.72	0.44
	After intervention	17.04	2.77	14.70	2.73	< 0.001
	Score change	2.13	2.35	0.03	0.27	
	*P*-value	< 0.001		0.322

**Table 5:** Distribution of the frequency of cues to action before intervention in two groups (intervention and control)

**Variable**	**Intervention group**		**Control group**	
	**Frequency**	**(%)**	**Frequency**	**(%)**
**Family**	42	72.4	42	73.7
**School**	10	17.2	9	15.8
**Relatives**	13	22.4	9	15.8
**Friends**	6	10.3	13	22.8
**Written media**	8	13.8	3	5.3
**Non-written media**	6	10.3	10	17.5
**Employees of Comprehensive Health Service Centers**	7	12.1	3	5.3

## Discussion

According to the results of the present study, the mean scores of puberty health behaviors increased significantly in the intervention group after the educational intervention, so that the female students' compliance with the following puberty health principles and tips increased: appropriate nutrition during menstruation, gentle exercise during menstruation, hygiene of the genital area, enough sleep and rest, bathing, purity after defecation, treatment of premenstrual and menstrual pains, acne care, timely replacement of menstrual pads and their proper disposal, and using underwear with right color and material. Consistent with our findings, there was significant improvements in puberty health behavior after education in studies [15, 17, 18]. Thus, HBM is a suitable useful model for creating health behaviors like health behavior during puberty, and it can be utilized in planning for adolescent health promotion in developing countries.

Our findings indicated that both research groups (intervention and control) had moderate levels of knowledge before the intervention probably due to the embarrassment and modesty of the family, the inflexibility of schools for puberty and menstruation, the lack of necessary educational programs in this field, and insufficient education in comprehensive health service centers. The difference in mean scores of students' knowledge in the second stage (one month after the intervention) might be due to the effect of the educational intervention on the students' active participation in educational classes. The findings were consistent with the results of previous studies [13, 16]; however, they were inconsistent with one study [19] Probably due to the difference in the implementation of educational programs, the research population and tools, and the traditional method of education, and the nonuse of health education models and theories. Knowledge about the health behaviors of puberty as a prerequisite lead to the creation of right attitudes and beliefs and the adoption of the right behavior.

Our findings indicated that the mean score of the perceived sensitivity construct was low before the educational intervention, and the students did not see themselves at risk of diseases caused by not performing the proper behavior during menstruation. The mean perceived sensitivity score of the intervention group increased significantly one month after the educational intervention. A number of studies [20–22] were consistent with the present study, demonstrating the efficiency of HBM in improving the perceived sensitivity in adolescents. Nevertheless, the studies were inconsistent with some studies [18, 23], owing to the short duration of education to change attitudes.

The higher mean score of the perceived severity of the intervention group after the educational intervention indicated that the individuals in this group considerably understood the risks of non-compliance with health tips during puberty and that if they did not follow the tips, they would be in extreme danger. Some studies [15, 24] were consistent with the present study; however, some studies [25, 26] were inconsistent with the present study owing to the small number of educational sessions and the lack of various educational methods in the studies.

Perceived benefits refer to individuals' perception of the positive effects and consequences of the recommended health behaviors during puberty, so that the educator seeks to affect it. Individuals in the intervention group gained a greater perception of the benefits of compliance with health tips during puberty. Some studies [24, 27] were consistent with the present study; however, study by Asadzandi et al. [28] was inconsistent with the present study probably due to the short intervention time. It is worth noting that an individual's perception of benefits paves the way for action, and there is a strong relationship between perceived benefits and adoption of preventive behavior.

Perceived barriers refer to an individual's mental perception of the financial and psychological costs of recommended behaviors regarding puberty health. Perceived barriers refer to potential negative factors of a certain health action that may inhibit the suggested behaviors [10]. Hence, an accurate perception of the barriers to performing health behaviors during puberty can encourage individuals to perform such behaviors as far as possible. In the present study, the reduction of perceived barriers in the intervention group was carried out by brainstorming, removing false beliefs, and providing solutions to overcome the barriers. Some studies [15, 24] were consistent with the current study.

In the present study, perceived self-efficacy increased significantly after the educational intervention. Self-efficacy refers to individuals' judgments about their confidence in the ability to perform specific actions [10]. After educational interventions, most students can comply with health tips during puberty such as proper nutrition during menstruation, importance of daily exercise, hygiene of the genital area, sufficient sleep and rest, bathing, purity after defecation, hygiene during menstruation, treatment of menstrual pain, and premenstrual syndrome. The findings were consistent with previous studies [18, 29]. Although in the control group, the difference in scores for constructs of perceived severity, benefits, barriers, and self-efficacy before and after the intervention was statistically significant, this difference was small. It could be due to reading the questionnaire and becoming sensitive to the questions.

In the present study, the highest frequency of acquiring information about menstruation was obtained through the family. Consistent with our findings, the family was the most important source of information in some studies [21, 28], since the home environment is the first locus of learning and teaching through different ways, particularly imitation, and children spend most of their lives in this place and copy models of their family members, especially their parents. In one study [15], the school was the most important source of information owing to the deprivation of family environment in girls living in boarding centers.

According to the obtained results, it is suggested that school health educators be trained to implement a similar pattern-based educational intervention based on the educational package of the above study in Rasht city, and then its results will be evaluated.

### Strengths of this research

Strengths of this research include that the educational intervention was based on the health belief model and used suitable methods for each structure, attracting the active participation of students.

### Research limitations

The research limitations included the students' unwillingness to participate in the study as well as the use of questionnaires and self-reporting. Some participants might refuse to give real answers.

## Conclusion

There were statistically significant differences among the mean scores of knowledge, the HBM constructs, and health behaviors during puberty in the intervention group after the educational intervention; however, there was no difference in the control group. According to the results, the health education program designed based on HBM in the field of observing the health points during puberty was efficient and effective. Obviously, the low frequency percentage of obtaining information through school can indicate the need to activate schools in the field of providing this kind of education to students, considering the unique roles of schools and teachers in education, and raising healthy and dynamic individuals guaranteeing the future of any country.

## Data Availability

All data generated during and/or analyzed during the study are available from the corresponding author on reasonable request.

## Abbreviations

HBM: Health Belief Model
CVR: Content Validity Ratio
CVI: Content Validity Index
